# Battery Energy Storage Sizing When Time of Use Pricing Is Applied

**DOI:** 10.1155/2014/906284

**Published:** 2014-09-11

**Authors:** Guido Carpinelli, Shahab Khormali, Fabio Mottola, Daniela Proto

**Affiliations:** ^1^Department of Electrical Engineering and Information Technology, University of Naples Federico II, Via Claudio, No. 21, 80125 Napoli, Italy; ^2^Department of Industrial and Information Engineering, Second University of Naples, Via Roma 29, 81031 Aversa, Italy

## Abstract

Battery energy storage systems (BESSs) are considered a key device to be introduced to actuate the smart grid paradigm. However, the most critical aspect related to the use of such device is its economic feasibility as it is a still developing technology characterized by high costs and limited life duration. Particularly, the sizing of BESSs must be performed in an optimized way in order to maximize the benefits related to their use. This paper presents a simple and quick closed form procedure for the sizing of BESSs in residential and industrial applications when time-of-use tariff schemes are applied. A sensitivity analysis is also performed to consider different perspectives in terms of life span and future costs.

## 1. Introduction

Minimizing the electricity costs is one of the greatest challenges related to the use of batteries in modern smart grids. Focusing on the end customer point of view, residential homes and small-/medium-sized industrial facilities are expected to actively modify their energy spending patterns by adopting battery systems and optimizing their consumption. Other advantages can be obtained by improving power quality and reliability [1–3]. The key barrier to the use of such devices remains their high cost as batteries are still quite expensive. However, looking at the longer-term, the technological development is expected to play an important role in both cost reduction and performance's improvement, thus making batteries increasingly competitive for these applications [4].

To evaluate the benefit of using batteries, several factors must be taken into account such as electricity rates, load profile, technical and economic constraints of the battery, and grid connection policies. All the aforementioned factors are considered in this paper that discusses the economic analysis effected for sizing a battery system with the aim of reducing the cost sustained for the energy consumptions. In more detail, the users are expected to modify their energy spending patterns by adopting battery systems and optimizing their consumption in the frame of the applied energy tariff schemes. Obviously, only dynamic pricing programs can be considered at this purpose, in particular, real time and time-of-use (TOU) pricing. Real time pricing reflects the actual wholesale energy market and can suffer from large price variations in narrow time bands; TOU tariffs are based on only two or three price levels. Even if real time pricing seems to have high potential in the highly automated grid of the next future, nowadays it has not been widely accepted or implemented, whereas TOU pricing has been used extensively [5].

In literature, the evaluation of the benefits related to the use of batteries under TOU tariff schemes usually referred to the problem of optimal sizing of the battery [6–8]. In [6], the sizing is based on the maximization of the economic benefit which is defined as the ratio between the annual electricity saved (i.e., the difference between the total annual electricity charge without and with battery system) and the capital cost of the battery system. In [7], different combinations of technologies and sizes of the battery system are analyzed. The comparative analysis is made by evaluating the return of investment function, which is defined as the ratio between the revenue of the battery system (i.e., the difference between the capital cost and the total profit) and its capital cost. In [8], the most beneficial battery combination (i.e., technology and size) was identified on the basis of a closed form inequality. In particular, to check the profitability of the battery system, the costs with and without battery were compared.

In this paper, a methodology is proposed to study the profitability of a battery system for a customer under TOU pricing. In particular, a simple closed form procedure is proposed to evaluate the size of the battery system which minimizes the total costs sustained by the customer. The proposed procedure is able to account for both the technical constraints of the battery and contractual agreements between the customer and the utility. In the numerical application, the methodology is applied with reference to both residential and small industrial customers and based on actual TOU tariffs. Some aspects that affect the profitability of the battery, such as technological limitations (e.g., the battery and converter efficiency) and economic barriers (e.g., capital cost and the rate of change of the cost), are discussed.

Compared to that proposed in [6], important technical constraints on the use of the battery were taken into account (e.g., the depth of discharge). The net present values of all the costs were also included in the economic analysis.

Compared to the proposal in [7], this paper includes the constraints related to the contractual agreements between the customer and utility which refer to both the periods in which the battery charges and those in which it discharges. These constraints are taken into account in [8] only with reference to the discharging stage. The importance of taking into account these constraints leads to the high influence they have on the amount of energy exchanged with the grid.

Moreover, unlike the sizing procedure proposed in this paper, in [6–8], the trend variation of the load profile along the years is not taken into account. This paper performs also a wide sensitivity analysis to consider different perspectives in terms of life span and future costs.

The reminder of the paper is organized as follows: in Section 2, an economic analysis is effected with reference to a customer's system including BESS. The closed form solution is presented in Section 3. In Section 4, the results of numerical applications are reported and discussed. Conclusions are drawn in Section 5.

## 2. Economic Analysis

In this section, an economic analysis is effected in order to evaluate the benefits achievable using BESSs in terms of reduction of costs related to the electricity consumption. The economic analysis is performed by considering a specific time period whose choice can be related to the lifecycle of the system where the BESS is installed.

The economic analysis considers all the costs related to the inclusion of the battery system in a residential area or in an industrial facility that hereinafter will be referred to as the customer. In both cases, it is assumed that it is not possible to sell energy to the grid. Then, the energy stored in the battery can be used only to supply the loads. In the case of industrial facility, it is also assumed that the inclusion of the BESS does not introduce any modifications in the manufacturing process and in the facility's infrastructures. To evaluate the total customer costs related to the BESS adoption, capital, maintenance, replacement, disposal, and energy costs have to be taken into account:

(1) $$\begin{array}{c} C_{\text{LCC}}=C_{0}+C_{\text{mt}}+C_{\text{rep}}+C_{\text{disp}}+C_{\text{en}}, \end{array}$$

where *C*_LCC_ is the life cycle cost (or total customer cost), *C*_0_ is the capital cost of the BESS, *C*_mt_ is the BESS maintenance costs, *C*_rep_ is the cost related to the replacement of the batteries, *C*_disp_ is the BESS disposal cost, and *C*_en_ is the energy cost. The maintenance, replacement, and energy costs refer to their sum over the specified time period in which the economic analysis is performed. For each year, their net present values are evaluated by means of

(2) $$\begin{array}{c} {C_{k}(n)}_{\text{npv}}=C_{k}\left(n\right)\frac{{\left(1+\alpha \right)}^{n-1}}{{\left(1+\beta \right)}^{n-1}}, \end{array}$$

where *C*_*k*_(*n*) is the cost (energy, maintenance, or replacement cost) related to year *n*, *C*_*k*_(*n*)_npv_ is its net present value, *β* is the assumed discount rate, and *α* is the effective rate of change assumed for the cost.

BESS* capital costs *(*C*_0_) in (1) include equipment purchase cost and installation cost. Both costs of the battery system *C*_batt_ and the converter *C*_conv_ are considered:

(3) $$\begin{array}{c} C_{0}=C_{\text{batt}}+C_{\text{conv}}. \end{array}$$

BESS* maintenance costs *(*C*_mt_) in (1) include corrective maintenance and preventive maintenance costs [9]. They can also be included as percentage of capital costs [10].

BESS* replacement costs *(*C*_rep_) have to be sustained for purchasing a new battery if the battery lifetime is lower than the time period considered in the economic analysis. The lifetime of the battery depends on the number of charging/discharging stages per day:

(4) $$\begin{array}{c} \text{Battery}\;\;\text{lifetime}=\frac{N_{\text{cycles}}}{365*\upsilon }, \end{array}$$

where *N*_cycles_ is the total number of charging/discharging cycles declared by the battery manufacturer and *υ* is the number of daily charging/discharging cycles.

With reference to the BESS* disposal costs *(*C*_disp_) in (1), it is assumed that they can take into account also the benefit derived from the recycling of the battery. This cost/benefit may vary depending on the country where the disposal is performed. Based on the expected development of recycling technology, disposal activity could also be assumed to represent a benefit rather than a cost [11].

Energy costs (*C*_en_) in (1) are the costs sustained by the BESS's owner (i.e., the customer) related to the energy consumption. They include both the purchase of the energy required to supply its loads and that required to charge the battery. The benefit in terms of reduction of these costs is related to the discharge of the battery to supply the loads during peak price periods. In the evaluation of the energy costs, both the variations of the price of energy due to the inflation and load variations caused by economic growth can be taken into account by imposing a percentage variation per year.

The energy costs *C*_en_ can be evaluated as follows:

(5) $$\begin{array}{c} C_{\text{en}}=C_{\text{load}}+C_{\text{ch}}-C_{\text{dch}}, \end{array}$$

where *C*_load_ is the total electricity cost required to supply the loads without considering the presence of the BESS, *C*_ch_ is the electricity cost sustained for charging the battery, and *C*_dch_ is that avoided by the customer as loads are supplied by the BESS. Both *C*_ch_ and *C*_dch_ depend on the energy tariff applied to the customer. Utilities usually propose different tariffs depending on the periods (season) of the year [5]. In the most general case, the cost required for supplying the loads for the *n*th year is given by

(6) $$\begin{array}{c} C_{\text{load}}\left(n\right)=\overset{n_{S}}{\underset{i=1}{\sum }}N_{i}\overset{}{\underset{T_{\text{day}}}{\int }}{\text{EC}}_{i,n}\left(t\right)p_{L,i,n}\left(t\right)dt, \end{array}$$

where *p*_*L*,*i*,*n*_(*t*) is the power requested by the loads at time *t* of a typical day of the *i*th season of the *n*th year, *n*_*s*_ is the number of the seasons provided by the energy tariff, *T*_day_ is the duration of the day, *N*_*i*_ is the number of days of the *i*th season, and EC_*i*,*n*_(*t*) is the price of energy (energy charge) at time *t* of the *i*th season of the *n*th year.

In order to gain economic advantage, the battery is allowed to charge during low price hours and it is allowed to discharge during the high price hours. Thus, the costs sustained for charging the battery (*C*_ch_) and the cost avoided by the consumer as loads are supplied by the BESS (*C*_dch_) can be evaluated, for the *n*th year, as follows:

(7) $$\begin{array}{c} C_{\text{dch}}\left(n\right)=\overset{n_{s}}{\underset{i=1}{\sum }}N_{i}\overset{}{\underset{\Omega_{\text{dch},i,n}}{\int }}{\text{EC}}_{i,n}\left(t\right)p_{B,i,n}\left(t\right)dt, \end{array}$$

(8) $$\begin{array}{c} C_{\text{ch}}\left(n\right)=\overset{n_{s}}{\underset{i=1}{\sum }}N_{i}\overset{}{\underset{\Omega_{\text{ch},i,n}}{\int }}{\text{EC}}_{i,n}\left(t\right)p_{B,i,n}\left(t\right)dt, \end{array}$$

where *p*_*B*,*i*,*n*_(*t*) is the absolute value of the power of the battery at time *t* of the *i*th season of the *n*th year and *Ω*_ch,*i*,*n*_ and *Ω*_dch,*i*,*n*_ are the sets of all the time intervals of the day, in which the battery is allowed to charge and discharge, respectively. Eventually, by substituting (5) into (1), the total customer cost will be

(9) $$\begin{array}{c} {C_{\text{LCC}}=C}_{0}+C_{\text{mt}}+C_{\text{rep}}+C_{\text{disp}}+C_{\text{load}}+C_{\text{ch}}-C_{\text{dch}}. \end{array}$$

## 3. BESS Sizing Procedure under TOU Pricing

The optimum value of the size of the battery is the one corresponding to the minimum total cost

(10) $$\begin{array}{c} \min\left(C_{\text{LCC}}\right), \end{array}$$

subject to the following constraints:

- the number of charging/discharging cycles per day has to be coherent with the specified tariff;
- a maximum depth of discharge is allowed;
- the energy discharged by the battery must be used only to supply the load (the customer is not allowed to sell energy to the grid);
- the power absorbed by the customer cannot exceed a maximum value imposed by the contractual agreement;
- the energy stored in the battery at the beginning and at the end of the day has to be the same.

The* minimization* problem (10) subject to the constraints (i)–(v) can be solved by means of a classical optimization algorithm [2, 3, 12]. However, in case of TOU tariff, a simplified closed form procedure can be used which makes the evaluation more simple and straightforward. The details of the proposed closed form approach are given in the following.

In order to meet constraint (i), the number of daily charging/discharging cycles has to be evaluated. When the TOU tariff is made by two price periods, that is, the on-peak hours (the hours of higher price) and the off-peak hours (the hours of lower price), it is trivial to allow the charging of the battery during the off-peak hours and the discharging during the on-peak hours. It follows also that, if this case is considered, the battery is subjected to only one charging/discharging cycle in 24 hours. This condition can be still valid in case of TOU tariff made by three price levels, that is, the tariffs that provide a specified price for the mid-peak hours. In fact, usually, mid-peak periods refer to few hours [5]; thus, a benefit can be achieved if the battery is idle during these hours.

In the following, only for the sake of simplicity and without loss of generality, it is supposed that TOU tariff does not vary along the years; hence, the index *n* in EC_*i*,*n*_(*t*) in (6)–(8) and in *Ω*_ch,*i*,*n*_ and *Ω*_dch,*i*,*n*_ in (7) and (8) can be neglected. With reference to *C*_dch_(*n*), relationship (7) reads

(11) $$\begin{array}{c} C_{\text{dch}}\left(n\right)=\overset{n_{S}}{\underset{i=1}{\sum }}N_{i}{\text{EC}}_{\text{dch},i}E_{\text{dch},i,n}\eta_{\text{dch}}, \end{array}$$

where EC_dch,*i*_ is the energy charge (i.e., the price for energy) during the on-peak hours, that is, in the time intervals included in *Ω*_dch,*i*_, *E*_dch,*i*,*n*_ is the daily energy supplied by the battery in the same time intervals, and *η*_dch_ is the BESS discharge efficiency.

With reference to *C*_ch_(*n*), relationship (8) reads

(12) $$\begin{array}{c} C_{\text{ch}}\left(n\right)=\overset{n_{S}}{\underset{i=1}{\sum }}N_{i}{\text{EC}}_{\text{ch},i}E_{\text{ch},i,n}\frac{1}{\eta_{\text{ch}}}, \end{array}$$

where EC_ch,*i*_ is the energy charge (i.e., the price for energy) during the off-peak hours, that is, the time intervals included in *Ω*_ch,*i*_, *E*_ch,*i*,*n*_ is the daily energy requested by the battery in the same time intervals, both evaluated at the *n*th year, and *η*_ch_ is the BESS charge efficiency. When the energy tariff is assumed to vary along the years, formulas (11) and (12) can be easily extended.

In order to calculate *E*_dch,*i*,*n*_ in (11) and *E*_ch,*i*,*n*_ in (12), the following considerations have to be made.

By taking into account the maximum allowable depth of discharge (constraint (ii)), the maximum amount of energy that can be theoretically charged/discharged from the battery is given by

(13) $$\begin{array}{c} E^{T}=E_{\text{size}}\frac{\delta }{100}, \end{array}$$

where *E* ^*T*^ is the amount of energy that can be theoretically charged/discharged from the battery, *E*_size_ is the size of the battery, and *δ* is the percentage value of the maximum depth of discharge of the battery [13].

Equation (13), however, cannot be directly included in (11) and (12), because it gives only the theoretical value of the daily energy available for charging/discharging, since, during the operation, the practical value of the energy that can be discharged is limited by constraint (iii) and the maximum energy that can be charged is limited by constraint (iv).

With reference to constraint (iii), if initially the energy limit imposed by (13) is not considered, *Ω*_dch,*i*_ (i.e., the interval in which the battery is allowed to discharge) includes two time periods:

- the first, *Ω*_1,*i*,*n*_, includes all the time intervals in which the maximum power that can be supplied by the battery is greater than the maximum power requested from the load (i.e., *Ω*_1,*i*,*n*_ = {*t* : *P*_*B*,max⁡_ > *p*_*L*,*i*,*n*_(*t*)});
- the second, *Ω*_2,*i*,*n*_, includes all the time intervals in which the power requested from the load is greater than or equal to the maximum power that can be supplied by the battery (i.e., *Ω*_2,*i*,*n*_ = {*t* : *P*_*B*,max⁡_ ≤ *p*_*L*,*i*,*n*_(*t*)}),

where *p*_*L*,*i*,*n*_(*t*) is the active power required by the load at time *t* of the *i*th season, *n*th year, and *P*_*B*,max⁡_ is the maximum power that can be supplied by the battery. Since the customer is not allowed to sell energy to the utility, during *Ω*_1,*i*,*n*_, the maximum energy that can be discharged by the battery is

(14) $$\begin{array}{c} E_{1,i,n}=\frac{1}{\eta_{\text{dch}}}\overset{}{\underset{\Omega_{1,i,n}}{\int }}p_{L,i,n}\left(t\right)dt, \end{array}$$

whereas, during *Ω*_2,*i*,*n*_, the maximum energy that can be discharged by the battery is

(15) $$\begin{array}{c} E_{2,i,n}=\frac{1}{\eta_{\text{dch}}}\overset{}{\underset{\Omega_{2,i,n}}{\int }}P_{B,\max}dt. \end{array}$$

The maximum energy that can be discharged during *Ω*_dch,*i*_ is then given by

(16) $$\begin{array}{c} {E_{\max,i,n}^{\text{dch}}=E}_{1,i,n}+E_{2,i,n}. \end{array}$$

With reference to constraint (iv), if initially the energy limit imposed by (13) is not considered, *Ω*_ch,*i*_ (i.e., the interval in which the battery is allowed to charge) includes two time periods:

- the first, *Ω*_3,*i*,*n*_, includes all the time intervals in which the sum of the power requested by the load and the maximum power that can be used by the battery for charging is lower than the maximum value of the power that can be imported from the grid which is imposed by the contractual agreement (i.e., *Ω*_3,*i*,*n*_ = {*t* : (*P*_*B*,max⁡_ + *p*_*L*,*i*,*n*_(*t*)) < *P*_max⁡_^ca^});
- the second, *Ω*_4,*i*,*n*_, includes all the time intervals in which the sum of the power requested by the load and the maximum power that can be used by the battery for charging is greater than or equal to the maximum value imposed by the contractual agreement (i.e., *Ω*_4,*i*,*n*_ = {*t* : (*P*_*B*,max⁡_ + *p*_*L*,*i*,*n*_(*t*)) ≥ *P*_max⁡_^ca^}),

where *P*_max⁡_^ca^ is the maximum value of the power that can be imported from the grid which is imposed by the contractual agreement. Since the customer is not allowed to absorb more than *P*_max⁡_^ca^ from the grid, the maximum energy that can be charged during *Ω*_3,*i*,*n*_ is

(17) $$\begin{array}{c} E_{3,i,n}=\eta_{\text{ch}}\overset{}{\underset{\Omega_{3,i,n}}{\int }}P_{B,\max}dt, \end{array}$$

and the maximum energy that can be charged during *Ω*_4,*i*,*n*_ is

(18) $$\begin{array}{c} E_{4,i,n}=\eta_{\text{ch}}\overset{}{\underset{\Omega_{4,i,n}}{\int }}\left(P_{\max}^{\text{ca}}-p_{L,i,n}\left(t\right)\right)dt. \end{array}$$

The maximum energy that can be charged during *Ω*_ch,*i*_ is then given by

(19) $$\begin{array}{c} {E_{\max,i,n}^{\text{ch}}=E}_{3,i,n}+E_{4,i,n}. \end{array}$$

Finally, based on the hypothesis that during the day the total energy charged and discharged is the same (constraint (v)) and considering now the energy limit given by (13), it follows that *E*_dch,*i*,*n*_ in (11) and *E*_ch,*i*,*n*_ in (12) are given by

(20) $$\begin{array}{c} E_{\text{dch},i,n}=E_{\text{ch},i,n}=\min\left\{E^{T},E_{\max,i,n}^{\text{dch}},E_{\max,i,n}^{\text{ch}}\right\}. \end{array}$$

The proposed procedure is then able to find the maximum energy that can be effectively charged/discharged while meeting the battery constraints as well as the utility constraints.

It is worth to note that, until the energy available for discharging is lower than that allowed by the utility constraint, the profit derived from the BESS increases while battery size increases. On the other hand, if the energy available for discharging is greater than that allowed by the utility constraint, the profit derived by the adoption of BESS becomes constant, while the size increases. Similar considerations can be effected when BESS charges and the maximum value of power imposed by the contractual agreement between the customer and the utility is taken into account.

The proposed procedure for the calculation of *C*_ch_ and *C*_dch_ (given by (11) and (12)) will be applied to different battery sizes; thus, for each of them, the total cost will be evaluated by means of (9). The optimum value of the size of the battery will be that corresponding to the minimum total costs.

## 4. Numerical Applications

**Figure 1:**
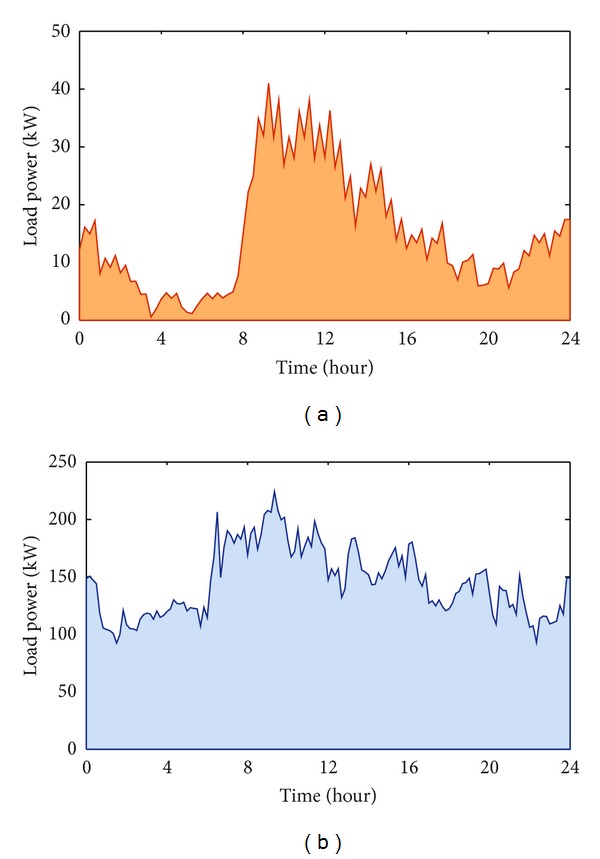
Residential load profile (a). Industrial load profile (b).

Two case studies are presented in which the proposed approach was performed with reference to two different load profiles that are reported in Figure 1: residential customer (Figure 1(a)) and industrial customer (Figure 1(b)). In the residential case, the data refer to customers of a common area of multifamily complexes, connected to an actual distribution network of South Italy through a point of common coupling [14]. In the industrial case, the data refer to a small industrial facility of South Italy [15]. The data were obtained either by direct measurements or by the electrical energy meters installed at the customers' feeding point.

For both of the residential and industrial loads, annual load variations of +1%, +3%, and +5% were considered, thus including different possible trends of load growth versus years.

Different combinations of the values assumed for the discount rate *β* and effective rate of change *α* were considered too, varying from 3% to 7% in order to evaluate different economic scenarios.

The BESS considered in this application includes a lithium-ion battery which is connected to the power grid by a PWM-controlled AC/DC converter. In order to take into consideration both the present technological status and the future perspectives, a sensitivity analysis was performed by considering for the BESS different charging and discharging efficiencies as well as different installation costs. The charging and discharging efficiencies were supposed to vary from 0.93 to 0.98 and from 0.96 to 0.98, respectively, whereas the installation cost was supposed to vary from 200 $/kWh to 1000 $/kWh. These costs include maintenance (2%) whereas the benefit deriving from the disposal of battery has been disregarded.

A maximum depth of discharge of 80% is assumed which corresponds to a life cycle of about 4500 cycles [4]. By imposing one charging/discharging cycle per day, a life cycle of 12 years can be assumed for the battery; replacement costs are not considered.

Regarding the energy rates, TOU tariff was considered with reference to the industrial and residential cases. For both of the two cases, the TOU tariff adopted by an actual utility was considered, whose values are reported in Tables 1 and 2 [16]. The considered tariff is applicable to both small and medium industrial customers as well as for service in common areas in multifamily complexes.

### 4.1. Case (a): Residential Load

**Figure 2:**
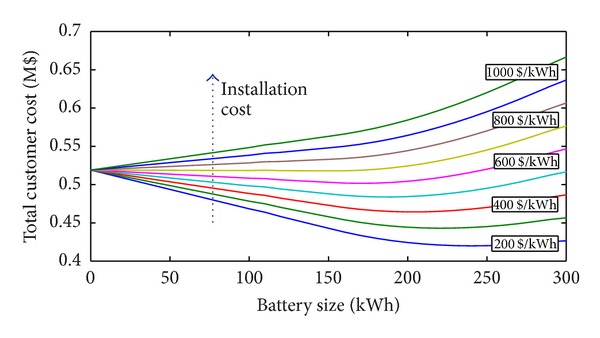
Total customer cost with *α* = 5%, *β* = 5%, *η*_ch_ = 95%, and *η*_dch_ = 98%, for 5% annual load variations and for different installation costs (residential load).

In this case study, the procedure was applied to a BESS of a residential load (Figure 1(a)). The total customer costs versus BESS sizes are reported in Figure 2 with reference to different BESS installation costs. The figure refers to the effective rate of change *α* = 5% and discount rate *β* = 5% and charge/discharge efficiencies of the BESS *η*_ch_ = 95%, *η*_dch_ = 98%, respectively. A 5% annual load variation was also supposed. In the figure, the benefit obtained for each size can be deduced. For a specific battery size, in fact, the difference between the total cost and the cost corresponding to size zero (if negative) represents the economic advantage (i.e., the benefit) obtained with the use of that battery. If the difference is positive, no convenience exists to install the BESS. This consideration applies also for the following figures.

In Figure 2, each curve is characterized by a minimum value of the total customer costs which corresponds to the optimal size of the BESS. Figure 2 shows that when the installation cost increases, the total cost increases as well and, at the same time, the optimal BESS size (if it exists) decreases. In addition, it is possible to observe that no benefits exist when the BESS installation cost is higher than 700 $/kWh. In these cases, in fact, the total costs are always greater than the case of size zero.

**Figure 3:**
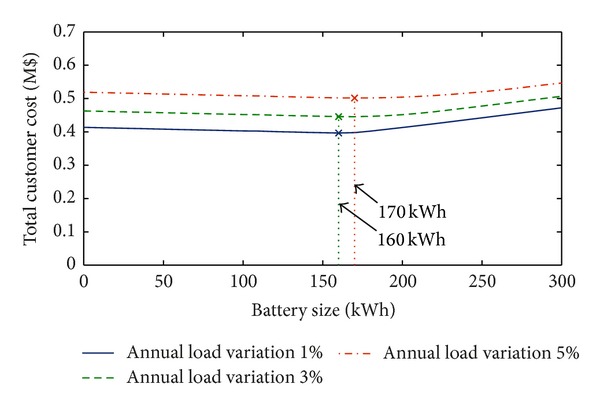
Total customer cost with *α* = 5%, *β* = 5%, *η*_ch_ = 95%, and *η*_dch_ = 98% for different values of annual load variation (residential load).

Focusing on the case of installation cost equal to 600 $/kWh, Figure 3 reports the results of the analysis performed with reference to different annual load variations. Also, in this case, *α* = 5%, *β* = 5%, *η*_ch_ = 95%, and *η*_dch_ = 98%. The figure shows that, as expected, the total cost increases when the annual load variation increases. The benefit provided by the use of BESS increases with the load variation and the optimal size of BESS increases from 160 kWh (for 1% and 3% annual load variations) to 170 kWh (5% annual load variation).

**Figure 4:**
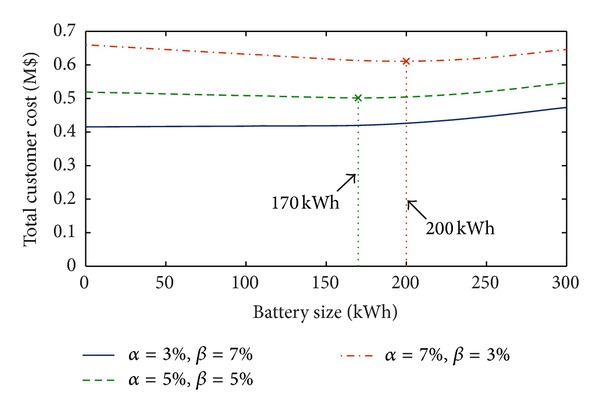
Total customer cost with an annual load variation of 5%, *η*_ch_ = 95%, and *η*_dch_ = 98% and for different values of *α* and *β* (residential load).

Figure 4 shows the total cost and optimal sizes of battery under different values of the effective rate of change *α* and discount rate *β* with the annual load variation of 5% and *η*_ch_ = 95% and *η*_dch_ = 98%.

In Figure 4, it is interesting to note that the total cost increases when *β* < *α*. On the other hand, when *β* > *α*, the total cost decreases and, in the particular case reported in the figure (*α* = 3%, *β* = 7%), the installation of BESS never provides any benefit, since the total cost for size zero is always lower than those obtained for all the other battery sizes. In case of *β* ≤ *α*, instead, a benefit is evidenced. Also, it can be observed that the values of *α* and *β* strongly affect the optimal size, since their variation results in very different values of total cost (up to 18%).

**Figure 5:**
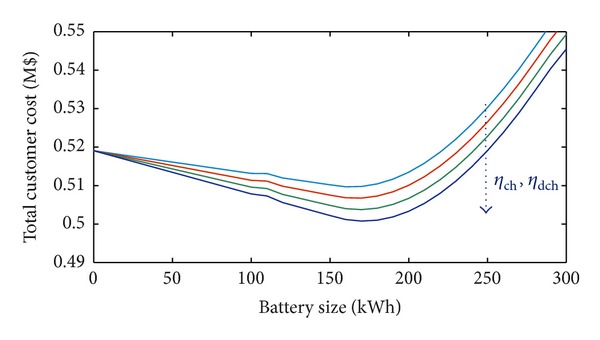
Total customer cost with *α* = 5%, *β* = 5%, and 5% annual load variation and different values of BESS efficiency (residential load).

In Figure 5, different BESS efficiencies are considered when the installation cost is 600 $/kWh, *α* = 5%, *β* = 5%, and the annual load variation is 5%. Obviously, the total cost decreases when efficiency increases, thus making the BESS even more profitable.

### 4.2. Case (b): Industrial Load

**Figure 6:**
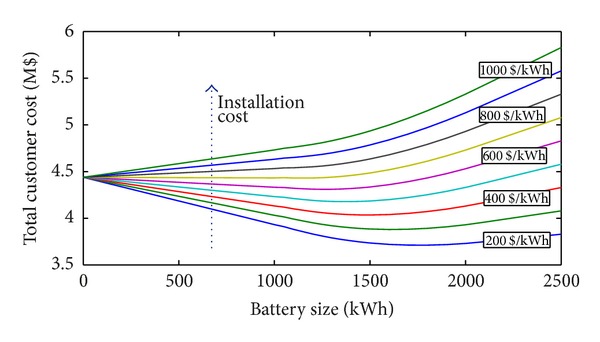
Total customer cost with *α* = 5%, *β* = 5%, *η*_ch_ = 95%, and *η*_dch_ = 98%, for 5% annual load variations (industrial load).

**Figure 7:**
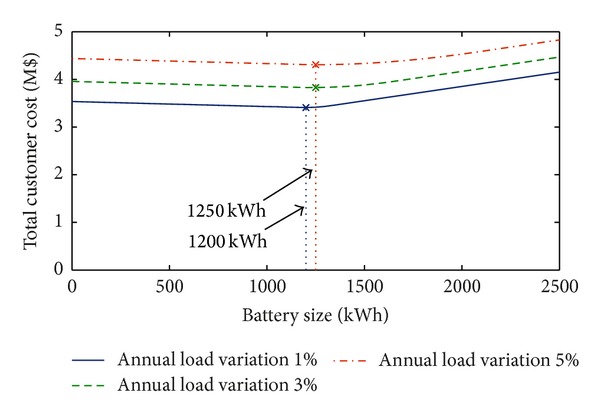
Total customer cost with *α* = 5%, *β* = 5%, *η*_ch_ = 95%, and *η*_dch_ = 98% for different values of annual load variation (industrial load).

In this case study, the power profile of a small industrial facility was considered (Figure 1(b)). In Figure 6, the results obtained for different installation costs are reported with *α* = 5%, *β* = 5%, *η*_ch_ = 95%, *η*_dch_ = 98%, and annual load variations of 5%. The total cost for different annual load variations is shown in Figure 7 when *α* = 5%, *β* = 5%, *η*_ch_ = 95%, *η*_dch_ = 98%, and the installation cost is 600 $/kWh.

Compared with those of Case (a), in both figures, the values of the total cost and benefits are larger (because the load power request is larger). However, the same considerations reported with reference to the residential case are still valid. In particular, from the analysis of Figure 6, it is very interesting to note that, even if the industrial load request is larger than the residential one, the maximum installation cost that makes the use of BESS profitable is about 700 $/kWh, that is, the same obtained in the residential case. As it can be observed in both figures (Figures 6 and 7), the optimal size of the battery increases with the load request. In the considered cases reported in Figure 7, the optimal size of the battery is 1200 or 1250 kWh, thus highlighting a slight influence of the annual load variation on the battery sizing.

**Figure 8:**
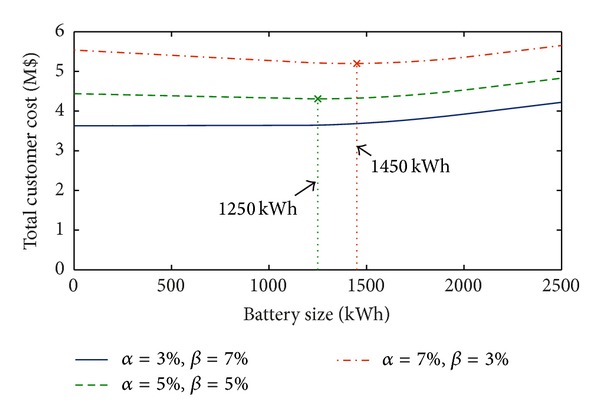
Total customer cost with an annual load variation of 5%, *η*_ch_ = 95%, and *η*_dch_ = 98% and for different values of *α* and *β* (industrial load).

Figure 8 shows the total cost and optimal sizes of battery under different values of the effective rate of change *α* and discount rate *β* based on annual load variation of 5%, *η*_ch_ = 95%, *η*_dch_ = 98%, and installation cost 600 $/kWh. Again, the same considerations reported with reference to the residential case (Figure 4) are still valid. In particular, Figure 8 shows that the total cost increases when *β* < *α* or decreases when *β* > *α* and, in the case reported in the figure (*α* = 3%, *β* = 7%), the installation of BESS never provides any benefit. Benefits are evidenced when *β* ≤ *α*. Also, it can be observed that the values of *α* and *β* strongly affect the optimal size, since their variation results in very different values of the total cost (up to 16%).

**Figure 9:**
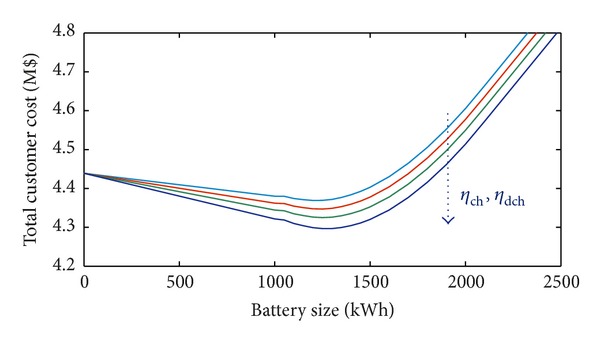
Total customer cost with *α* = 5%, *β* = 5%, and 5% annual load variation and different values of BESS efficiency (industrial load).

Also, in Figure 9, where different BESS efficiencies are considered, based on installation cost of 600 $/kWh, *α* = 5%, *β* = 5%, and annual load variation of 5%, the same considerations on the profitability of BESS can be drawn. Figure 9, in fact, shows that the total cost decreases when efficiency increases.

## 5. Conclusions

In this paper, a simplified procedure aimed at sizing BESSs was proposed. At this aim, a closed form procedure was presented under TOU tariff pricing schemes. A sensitivity analysis was performed on the basis of the variation of some parameters that affect the profitability of BESSs. By the analyses performed, it emerged that the parameters that mainly determine the profitability of BESSs are the “on-peak/off-peak” price variations, the installation costs, and financial parameters (i.e., effective rates of change and discount rates). BESS efficiency and annual load variation slightly contribute to the increase or decrease in the benefit.

The main outcome of the performed analysis is that the significance of the benefit achievable by using storage systems is mainly related to two aspects:

- the need to reduce installation costs which are still quite high,
- the energy tariff which should be characterized by a larger spread between on-peak and off-peak prices.

Regarding the first aspect, the technical research assumes an important role in order to find technological solutions which reduce manufacturing costs and prolong the battery lifetime. With reference to the second aspect, more incentivizing tariffs should be applied in order to justify the use of storage systems, thus increasing the number of potential demand response providers.

## Figures and Tables

**Table 1:** TOU tariff periods.

Summer tariff	
On-peak	12:00 noon to 6:00 pm
Part peak	8:30 am to 12:00 noon and 6:00 pm to 9:30 pm
Off-peak	9:30 pm to 8:30 am

Winter tariff	

Part peak	8:30 am to 9:30 pm
Off-peak	9:30 pm to 8:30 am

**Table 2:** TOU tariff prices.

TOU periods	Summer tariff ($/MWh)	Winter tariff ($/MWh)
On-peak	542.04	161.96
Part peak	252.90	
Off-peak	142.54	132.54
